# A Case Report on Multiple Sclerosis Associated With Atrial Fibrillation and Neurogenic Hypertension: Area Postrema Syndrome?

**DOI:** 10.7759/cureus.55860

**Published:** 2024-03-09

**Authors:** David Prentice, Ravi Ambati

**Affiliations:** 1 Neurosciences, Perron Institute for Neurological and Translational Science, Perth, AUS; 2 Neurology, Royal Perth Hospital, Perth, AUS

**Keywords:** atrial fibrillation, area postrema (ap), bell's palsy, neurogenic hypertension, multiple sclerosis

## Abstract

Multiple sclerosis (MS) is an autoimmune demyelinating neurological disorder primarily manifesting with a range of neurological symptoms, with cardiovascular autonomic involvement being a rare occurrence. We report a case where a patient initially presented with Bell's palsy, without other notable symptoms or signs, and subsequently developed atrial fibrillation, hypertension, and hemiparesis. Magnetic resonance imaging (MRI) revealed extensive demyelination in the cerebral hemispheres, brainstem, and notably, the area postrema. The anatomy of the area postrema and its connections, in relation to neurogenic hypertension, are discussed. The demyelination in the area postrema was thought to be the cause of our patient’s arrhythmias and acute hypertension. Furthermore, we discuss the cerebral origins of cardiac arrhythmias, with a focus on MS and other neurological conditions. This case underscores the rarity of isolated cranial neuropathies, such as Bell's palsy, as an initial sign of MS, marking the onset of a relapse.

## Introduction

Multiple sclerosis (MS) is a chronic autoimmune, demyelinating neurological condition that presents with various neurological syndromes, including optic neuritis, myelitis, internuclear ophthalmoplegia, trigeminal neuritis, and ataxia. The incidence is increasing, with the current prevalence in America being 1 in 300 persons. Diagnosis is confirmed by brain and spinal cord MRIs showing characteristic lesions, as well as the presence of oligoclonal bands in the cerebrospinal fluid (in over 80% of patients). Radiological features highly suggestive of MS include periventricular (in direct contact with the ventricles) or juxtacortical T2-weighted white matter lesions greater than 3 mm, ovoid or round lesions with a central vein sign, T2-weighted lesions perpendicular to the corpus callosum (Dawson's fingers), and short segment myelitis sparing the grey matter. Infratentorial lesions, which are common and patchy, often involve the periphery of the pons and medulla along with the nerve root entry zones of the trigeminal nerve. The recognized clinical forms of MS are relapsing-remitting, primary progressive, secondary progressive, and benign MS. Current treatments include interferons, teriflunomide, natalizumab, and ocrelizumab. Recently, autoreactive B cells induced by infectious mononucleosis have been thought to be central to the pathogenesis of MS [1].

The differential diagnosis of MS includes neuromyelitis optica (NMO), myelin oligodendrocyte glycoprotein antibody-associated disease (MOGAD), CNS vasculitis, sarcoidosis, systemic lupus erythematosus, Sjögren’s syndrome, and Behçet’s disease. NMO and MOGAD are important mimics of MS, as optic neuritis and myelitis are common presenting symptoms. NMO is caused by aquaporin 4 antibodies, with aquaporin 4 being the major water channel in the astrocyte foot processes. NMO presents with severe optic neuritis, often bilateral and sequential, with long posterior optic nerve involvement. The spinal cord disease presents as long, extensive transverse myelitis over three or more vertebral bodies, resulting in para- or tetraplegia. A unique early symptom is nausea, vomiting, and hiccups due to area postrema (AP) involvement. MOGAD is an autoimmune disease against oligodendrocytes and, therefore, myelin; it also causes severe optic neuritis (affecting the bulbar segment of the optic nerve) and extensive longitudinal myelitis. The diagnosis is made by detecting anti-myelin oligodendrocyte glycoprotein (MOG) in the serum or CSF [1].

Our patient’s presentation was unusual with Bell’s palsy as the first relapse of his MS and severe autonomic disturbance (hypertension and atrial fibrillation) heralding the second relapse. Although cerebral lesions have been recognized as a cause of cardiovascular symptoms ( arrhythmias, pulmonary edema, myocardial stunning (Takotsubo cardiomyopathy), myocardial necrosis, and autonomic dysfunction), brainstem MS as a cause is exceedingly rare [2]. The MRI revealed the area postrema was affected, which is usually only seen in NMO or glial fibrillary acidic protein encephalitis (GFAP) [3,4]. Interestingly our patient did not experience nausea, vomiting, or hiccups perhaps due to the lack of extensive AP involvement and high cervical cord involvement, which is often seen in NMO and GFAP encephalitis [3,4].

## Case presentation

A Caucasian man in his 30s with a history of classical migraine initially presented with a sudden onset of left facial palsy. He reported numbness of the upper and lower lips 24 hours prior to the facial weakness and some left auricular pain. Examination showed complete Bell's palsy with loss of taste on the anterior two-thirds of the tongue and left hyperacusis. No skin, aural, or mucosal lesions consistent with Ramsey-Hunt syndrome were noted. The rest of the neurological examination was normal, with normal visual acuity, visual fields, and fundal findings. His initial treatment with prednisolone was discontinued due to the adverse effects of psychosis and irritability, without improvement in his facial paralysis. Four months after the onset of Bell's palsy, he noticed a worsening of his facial droop.

Five months later, he complained of a sudden onset of severe headache, diaphoresis, and palpitations. Upon arrival in the emergency department, his blood pressure was 200/140 mmHg, with rapid atrial fibrillation (AF)/supraventricular tachycardia and a heart rate of 120-180 beats per minute. Treatment with adenosine was ineffective, and while verapamil controlled his heart rate, it did not restore normal sinus rhythm. However, by the next morning, he spontaneously reverted to a normal sinus rhythm with a pulse rate of 85 beats per minute and a blood pressure of 100/80 mmHg.

Neurological examination revealed mild bilateral optic atrophy, fine horizontal nystagmus on left and right horizontal gaze, vertical nystagmus on upward gaze, mild left lower motor neuron pattern facial weakness, left hemianesthesia (face > arm > leg), symmetrical bilateral brisk reflexes, mild gait ataxia (tendency to fall to the right), and a negative Romberg sign.

**Figure 1:**
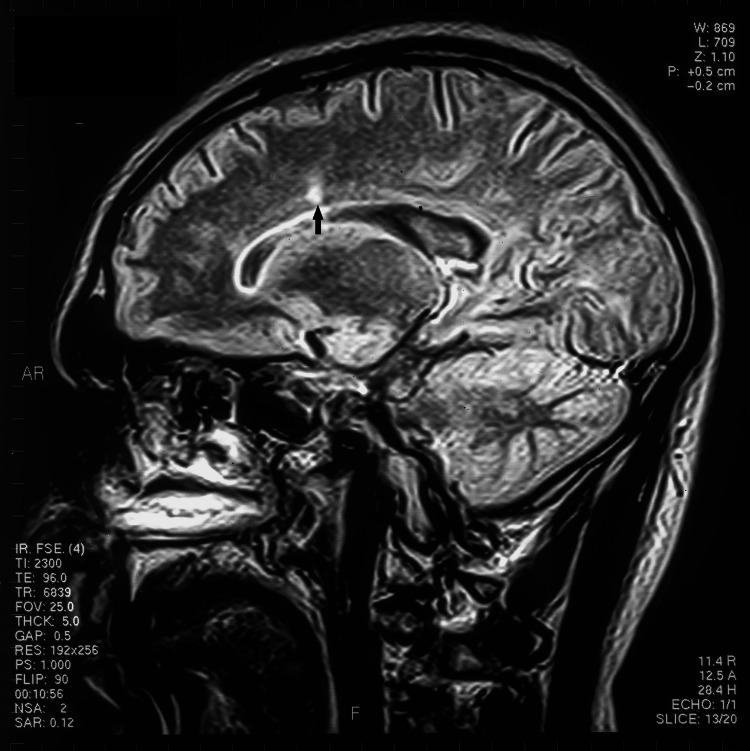
T1 sagittal MRI arrow showing an area of demyelination adjacent to the corpus callosum

**Figure 2:**
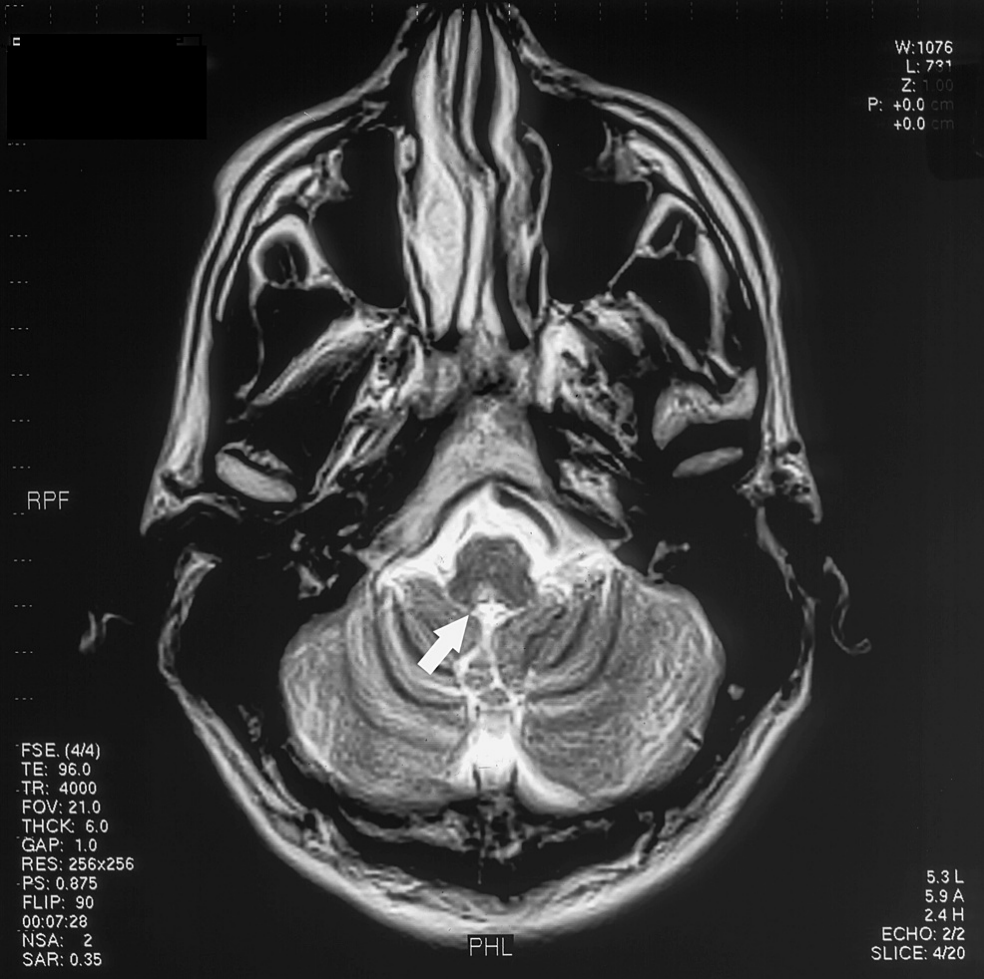
1T MRI axial Image showing an area of demyelination in the right medulla on the floor of the fourth ventricle (white arrow)

**Figure 3:**
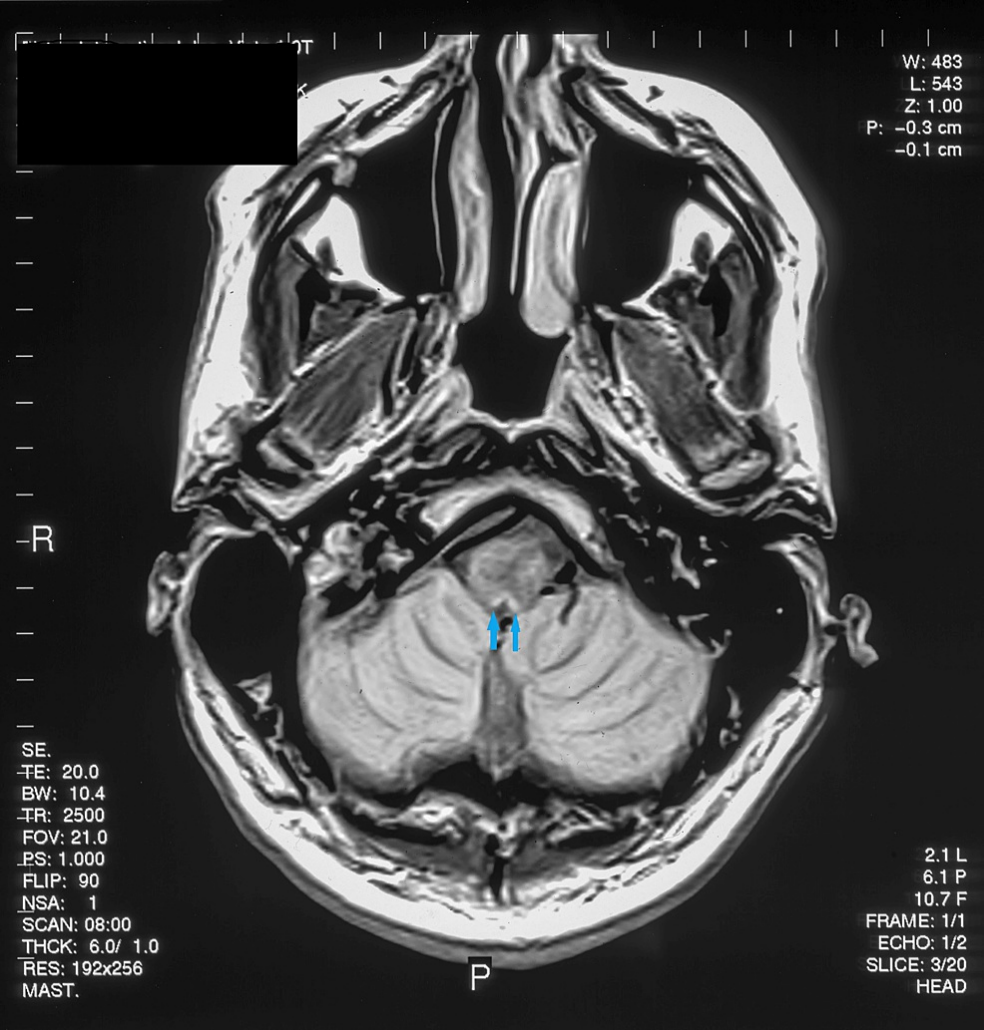
1T axial MRI T2-weighted image showing enhancement of area postrema bilaterally (blue arrows)

The preliminary diagnosis was a brainstem stroke or a space-occupying lesion, and further investigations were conducted. A CT brain scan was normal. Cerebrospinal fluid analysis (Table 1) showed a normal protein level (0.43 g/L) and positive oligoclonal bands while glucose levels (3.4 mmol/L) and cell counts were normal (leukocytes 4 x 10^6/L, erythrocytes 16 x 10^6/L). Autoimmune screenings, including antinuclear antibody (ANA) and antineutrophilic cytoplasmic antibody (ANCA), were negative, but angiotensin-converting enzyme (ACE) levels were mildly elevated at 54. Urine tests for catecholamines were negative. Visual-evoked potential tests indicated abnormalities in the left anterior visual pathway. Brain MRI fluid-attenuated inversion recovery (FLAIR) and T1 axial sequences showed enhancement of the area postrema bilaterally and lesions of the right medulla, as well as corpus callosal lesions typical of multiple sclerosis (Figures 1-3).

**Table 1:** Cerebrospinal fluid findings and serum autoantibodies ANA: antinuclear antibody; ANCA: antineutrophilic cytoplasmic antibody

	Patient	Normal Values
Cell Count WBC	4 x 106/L WBC	0-5 106/L WBC
Cell Count RBC	16 x 106/L	nil
Protein	0.43 g/l	0.18-0.45 g/l
Glucose	3.4 mmol/l	2.5-3.5 mmol/l
Oligoclonal Bands	positive	negative
Antibodies ANA and ANCA	negative	negative

Our patient qualifies for the diagnosis of MS based on the 2017 McDonald’s criteria ( 2 clinical attacks with > 2 MRI lesions) and the magnetic resonance imaging in multiple sclerosis (MAGNIMS) with dissemination in space > 3 periventricular lesions and > 1 infratentorial lesions.

His current treatment regimen includes teriflunomide 14 mg daily in the morning, baclofen (10 mg in the morning, 20 mg at lunch, 5 mg at night), pregabalin 150 mg twice daily, escitalopram 20 mg twice daily, and propranolol 40 mg twice daily. He has only been hospitalized once since then, with severe left leg cramps, and was commenced on baclofen. His current disabilities include mild left facial and leg paresis with left leg neuropathic pain. Follow-up MRIs have shown stable disease with no new plaques.

## Discussion

Paroxysmal atrial fibrillation has been documented in only two published cases of MS relapses. Chagnac et al. described a 37-year-old female who presented with vertigo, headache, and diplopia [5]. Upon examination, she had right facial palsy, internuclear ophthalmoplegia, and left hemiplegia. She subsequently went into rapid atrial fibrillation, which reverted with intravenous digoxin. MRI revealed a right pontine lesion and multiple supratentorial lesions consistent with MS. Schroth et al. documented a young female complaining of headache, vertigo, ataxia, and left facial numbness [6]. Her MRI showed a demyelinating plaque involving the left middle and inferior cerebellar peduncles and extending into the brainstem. She developed atrial fibrillation and pulmonary edema despite the relatively slow rate of AF. Treatment with intravenous digoxin and diuretics reverted her to sinus rhythm. Paroxysmal atrial fibrillation following high-dose corticosteroids for an MS relapse has also been described [7]. This is thought to be a direct effect on the myocyte potassium channels.

Autonomic abnormalities are recognized in patients with MS [8]. In an age-, sex-, and comorbidity-matched group study, 84 MS patients were compared to controls, revealing an increase in P wave duration and dispersion in the MS group [9]. Patients with idiopathic paroxysmal atrial fibrillation have similar ECG abnormalities, which are predictive of AF recurrence [9]. Paradoxically, the incidence of AF is decreased in MS populations due to a lower incidence of valvular heart disease and hypertension [10,11].

Cardiac arrhythmias (atrial and ventricular) occur with both supratentorial and infratentorial cerebral lesions. Cerebral infarction, hemorrhage, tumors, and subdural hematomas have been implicated. Specific areas of the brain highly associated with cardiac arrhythmias are the insular cortex and brainstem. Insular stroke and hemorrhage are linked with a new onset of atrial fibrillation [2]. The area postrema is an important area for autonomic function, with dysfunction of this area causing nausea, vomiting, hypertension, pulmonary edema (Takotsubo and stunned heart cardiomyopathy), and anorexia [12]. This area is anatomically close to and connected with the dorsal vagal nuclei, tractus solitarius, and nucleus ambiguus. An increase in vagal tone leads to inhomogeneous atrial repolarization with subsequent atrial fibrillation. Increased sympathetic activation causes hypertension, tachycardia, and myocardial stunning via subendocardial ischemia and necrosis. In both our patient and the previous cases, acute demyelination occurred in this region. Although MS is rarely associated with "area postrema syndrome" (nausea, vomiting, hiccups, and anorexia), neuromyelitis optica (NMO) [3] and glial fibrillary acidic protein (GFAP) encephalitis [4] can present with this syndrome. The area postrema is on the floor of the fourth ventricle dorsal to the vagal nucleus and is a bilobed structure (Figures 2, 3) [13]. In our patient, this area is involved in demyelination, together with plaques in the pons and supratentorial white matter typical of MS (Figure 3). Testing for GFAP, Aquaporin 4, and MOG antibodies was not available at the time of presentation.

Pure cranial neuropathies are rarely a presenting feature of MS, as most patients exhibit a combination of brainstem and cerebellar signs. A Bell's palsy presentation, as in our patient, is rare in MS; however, one study reported it occurred in 4.7% of cases as a first symptom in 107 MS patients [14]. The poor response to steroids may have provided an important clue. MRI is not routinely performed for cases of presumed Bell's palsy. A patient with Ramsey Hunt syndrome developed transient atrial fibrillation, which was attributed to vagal nerve involvement [15].

Although tests for MOG, Aquaporin 4, and GFAP antibodies were not performed, our patient had the typical clinical and radiological features of MS. He fulfilled the McDonald criteria [16], with lesions and symptoms disseminated in time and space, along with CSF oligoclonal bands. MRI demonstrated typical Dawson's fingers and periventricular white matter lesions. We recognize that NMO and MS can overlap, but in this case, the diagnosis is compatible with MS. Rare cases of MS present with APS or have isolated MRI area postrema enhancement. Koh et al. documented a 36-year-old with known MS who presented with intractable vomiting as an initial symptom of her relapse [17]. She tested negative for Aquaporin-4 and MOG antibodies and had MRI enhancement of the area postrema. Birkhead and Friedman reported a patient whose first symptoms of MS were hiccups and vomiting [18]. It had previously been thought these MRI lesions were unique to NMO but are also found in GFAP MOG and Bickerstaff encephalitis.

Lesions of the area postrema have been used as animal models of neurogenic hypertension [19]. The AP has a high density of angiotensin 2 receptors, and increased sympathetic activation through these receptors may contribute to neurogenic hypertension. Ablation of the AP in rats is often lethal, leading to pulmonary edema due to excessive sympathetic stimulation. Cases of MS have presented with neurogenic pulmonary edema [20] or Takutsubo’s cardiopathy [10] due to medullary lesions encompassing the AP. The cerebral origin of neurogenic pulmonary edema is debated, but most believe that lesions in the hypothalamus or the medulla are causative. Interestingly, subarachnoid hemorrhage with the release of blood products to the hypothalamus and AP (both have reduced blood-brain barriers) can cause stimulation (sympathetic) followed by damage (to endocrine and blood pressure control). We hypothesize that our patient’s arrhythmia and hypertension were a direct result of demyelination of the AP and brainstem nuclei.

## Conclusions

We hypothesize that our patient’s arrhythmia and hypertension were a direct result of demyelination of the AP and brainstem nuclei. Recognition of the cerebral origin of myocardial dysfunction and arrhythmia may be delayed or inapparent. A good history and examination, together with imaging, will help reveal the true origin of these events. The importance of autonomic networks of the insular and the area postrema is not widely appreciated. Area postrema syndrome whilst rare in MS is an important feature of neuromyelitis optica. Hypertension due to brainstem dysfunction is uncommon.
